# The outcomes of robotic ileocolic resection in Crohn’s disease compared with laparoscopic and open surgery: a meta-analysis and systematic review

**DOI:** 10.1007/s10151-025-03116-4

**Published:** 2025-03-26

**Authors:** M. Flaifel, S. Eichenberg, B. Mohandes, E. Taha, L. Kollmann, S. Flemming, A. Haberstroh, N. Ortlieb, N. Melling, K. Neumann, S. Taha-Mehlitz, T. Poškus, D. M. Frey, P. C. Cattin, A. Taha, J. Zeindler, R. Rosenberg, B. Saad, M. D. Honaker

**Affiliations:** 1 School of Medicine, St. George’s University of London, London, UK; 2 Department of Visceral Surgery, Cantonal Hospital Baselland, Basel, Switzerland; 3 Department of General Surgery, Mohammed Bin Rashid University of Medicine and Health Sciences, Dubai, UAE; 4 Department of Visceral, Gynecology and Pediatric Surgery, Al Qassim Health Cluster, Buraydah, Kingdom of Saudi Arabia; 5 Department for General, Visceral, Transplant, Vascular and Pediatric Surgery, University Hospital Würzburg, Würzburg, Germany; 6 Laupus Health Sciences Library, East Carolina University, Greenville, NC USA; 7 Data Science and Statistics, Medoc Swiss, Basel, Switzerland; 8 Department of General, Visceral and Thoracic Surgery, University Medical Center Hamburg-Eppendorf, Hamburg, Germany; 9 Department of Surgery, Dalhousie University, Halifax, NS Canada; 10 Clarunis, Department of Visceral Surgery, University Centre for Gastrointestinal and Liver Diseases, St. Clara Hospital and University Hospital Basel, Basel, Switzerland; 11 Institute of Clinical Medicine, Vilnius University Hospital, Vilnius, Lithuania; 12 Department of Surgery, Cantonal Hospital Baden, Baden, Switzerland; 13 Department of Biomedical Engineering, Faculty of Medicine, University of Basel, Allschwil, Switzerland; 14 Department of Surgery, Brody School of Medicine, East Carolina University, Greenville, NC USA; 15 Department of Acute Medicine, Royal Lancaster Infirmary, Lancaster, UK

**Keywords:** Ileocolic resection, Crohn’s disease, Robotic surgery, Laparoscopic surgery, Meta-analysis

## Abstract

**Background:**

This is the first review providing insights into the outcomes of robotic ileocolic resection for Crohn’s disease, potentially guiding improved surgical decisions and patient outcomes and comparing outcomes with laparoscopic and open approaches.

**Methods:**

The review was registered prospectively with PROSPERO (CRD42024504839). A comprehensive search of MEDLINE, Embase, Scopus, and Cochrane Central databases for studies on robotic ileocolic resection for Crohn’s disease from inception to February 2024 was conducted. Eligible studies included participants over 18 years of age with Crohn’s disease undergoing robotic ileocolic resection. Data were extracted according to PRISMA guidelines. For single-arm analyses, the random-effects model was used, while two-arm analyses employed the inverse variance and Mantel–Haenszel methods.

**Results:**

The analysis included eight studies with 5760 patients, among whom 369 underwent robotic ileocolic resection. The mean operative time for robotic procedures was 226 min. Postoperative complications included ileus in 12.50% and wound complications in 7.00%, while reoperations and readmissions occurred in 3.60% and 13.20% of patients, respectively. When compared with laparoscopic procedures, robotic procedures showed shorter length of hospital stay and longer operative times but similar total complication, reoperation, and conversion rates. In contrast, robotic procedures had fewer total postoperative complications compared with open surgeries, despite longer operative times.

**Conclusions:**

Robotic ileocolic resection for Crohn’s disease, while having a longer operative time, results in fewer postoperative complications compared with open surgery and shows comparable outcomes to laparoscopic procedures with shorter hospital stays.

**Supplementary Information:**

The online version contains supplementary material available at 10.1007/s10151-025-03116-4.

## Introduction

Crohn’s disease is a chronic inflammatory bowel disease (IBD) affecting millions of people worldwide [1]. Management typically involves medical therapy to control inflammation, including corticosteroids and immunomodulators. However, approximately 80% of patients with Crohn’s disease will require surgery in their lifetime owing to failure of treatment or disease complications [2].

Although open surgery has been the standard approach, minimally invasive procedures, such as laparoscopic surgery, have become increasingly popular owing to their advantages in terms of recovery and cosmetic results [3]. In addition, over the last decade, innovations in surgical techniques, particularly the development and use of the robotic platform, have led to studies evaluating this approach’s potential risks and benefits over more conventional laparoscopic methods [4].

Currently, the laparoscopic approach is considered to be the preferred surgical technique for performing ileocolic resection in Crohn’s disease, as it has been shown to result in fewer postoperative complications and quicker recovery times [4]. However, there is still controversy regarding the application of the laparoscopic approach for recurrent Crohn’s disease owing to a prolonged operation time and a higher incidence of conversion to open technique [5]. Recently, with the advancement of robotic techniques in areas of colorectal surgery, several studies have assessed the use of robotic ileocolic resection in treating Crohn’s disease, exploring whether it offers advantages over laparoscopic or open surgery.

The aim of this systematic review and meta-analysis is to evaluate and directly compare the outcomes of robotic, laparoscopic, and open ileocolic resections in Crohn’s disease. To our knowledge, no prior meta-analysis has examined all three modalities—open, laparoscopic, and robotic—of resection for the treatment of ileocolonic Crohn’s disease.

## Methods

### Search strategy and data sources

A comprehensive search of MEDLINE (PubMed), Embase (Elsevier), Scopus (Elsevier), and Cochrane Central (Ovid) databases, from inception to February 2024, was conducted. The search strategy, designed and conducted by a medical reference librarian (A.H.), involved keywords and controlled vocabulary for concepts including “robot assisted surgeries,” “minimally invasive surgeries,” “colectomy,” “ileocolic resection,” “Crohn’s disease,” and “inflammatory bowel disease.” In addition, the reviewers manually searched relevant references and bibliographies from the screened studies to ensure all potentially relevant studies were identified. The review was registered prospectively with PROSPERO (CRD42024504839). Database results were uploaded into Covidence review software where deduplication took place. Two reviewers (E.T. and B.M.) independently screened titles, abstracts, and full texts against the predefined eligibility criteria per Cochrane systematic review guidelines. Conflicts were resolved by an independent third reviewer (M.F.). The PRISMA checklist is presented in Supplementary Item 1.

### Eligibility criteria and quality assessment

Eligible studies must have met all the following criteria according to the PICO(S) framework: participants older than 18 years with Crohn’s disease undergoing ileocolic resection and comparative studies including participants undergoing robotic, laparoscopic, or open approaches were included where applicable. Outcomes of interest included primary clinical outcomes, surgical recurrence, and complications/adverse events following the procedure. Eligible study designs included randomized control trials, prospective and retrospective cohort studies, and case series. Case reports, abstracts, poster presentations, non-English language publications, and published literature that have considerable overlap between authors, centers, or patient cohorts were excluded. The methodological quality of each study was independently evaluated by two authors using the Risk of Bias in Non-randomized Studies of Interventions (ROBINS-I) tool for nonrandomized studies [6]. The ROBINS-I tool was used to evaluate the risk of bias across several domains, including study design, confounding variables, participant selection, intervention classification, deviations from planned interventions, missing data, outcome measurement, and the selection of reported results. Studies were classified as having a high risk of bias if they exhibited substantial confounding, inadequate adjustment for covariates, selective reporting of outcomes, or high levels of missing data without sufficient justification.

### Statistical analysis

Data extraction was conducted independently by two reviewers using a standardized data extraction form. Extracted information included study characteristics (authors, year, location, and design), population demographics, surgical approach details, comparator arms, clinical outcomes, and complications. Discrepancies were resolved through discussion or consultation with a third reviewer. The extracted data were entered into a structured database for analysis. For single-arm analyses, means of continuous variables and rates of binary variables were pooled using the random-effects model, a generic inverse variance method of Der Simonian, Laird [7]. Proportions underwent logit transformation prior to meta-analysis. For two-arm analyses, pooled means and proportions were analyzed using an inverse variance method for continuous data [8] and the Mantel–Haenszel method for dichotomous data [9]. The weight of each study was assigned on the basis of its variance. The heterogeneity of effect size estimates across the studies was quantified using the *Q* statistic and the *I*^2^ index (*P* < 0.10 was considered significant) [10]. A value of *I*^2^ of 0–25% indicates minimal heterogeneity, 26–50% moderate heterogeneity, and 51–100% substantial heterogeneity. Furthermore, a leave-one-out sensitivity analysis was conducted to assess each study’s influence on the pooled estimate by omitting one study at a time and recalculating the combined estimates for the remaining studies. Funnel plots were generated to assess publication bias. Data analysis was performed using Open Meta analyst software (CEBM, Brown University, Providence, RI, USA) for single-arm analyses and RevMan software version 5.4 Review Manager (RevMan; computer program; the Cochrane Collaboration, 2020, Copenhagen, Denmark) for two-arm analyses. If the mean or standard deviation (SD) were unavailable, the median was converted to the mean; the range, interquartile range, or confidence intervals were converted to the SD using the formulas from the Cochrane Handbook for Systematic Reviews of Interventions [8].

### Endpoints

The primary endpoints for this study were as follows. Surgical complications were reported to include postoperative ileus, wound complications, anastomotic leakage, etc. Postoperative ileus is defined as the need for nasogastric tube insertion or no bowel movement or flatus for more than 5 days. Intraoperative complications were reported to include intraoperative bowel injury and unanticipated intraoperative bleeding. Other complications evaluated included *Clostridium difficile* (*C. diff*) infection, anemia requiring transfusion, stoma-related complications, intestinal obstruction, and intra-abdominal abscess. Medical complications were reported to include urinary tract infection, acute kidney injury, acute urinary retention, pneumonia, deep vein thrombosis, dehydration, cardiac complications, etc.

The secondary endpoints for this study were as follows. The behavioral characteristics of Crohn’s disease were defined using the Montreal classification for Crohn’s disease where nonstricturing, nonpenetrating disease is defined as B1, stricturing disease is defined as B2, penetrating disease is defined as B3, and perianal disease is defined as P [11]. Open ileocolic resection was defined as a procedure requiring a full abdominal incision. Reoperation rate was defined as the proportion of patients who underwent a subsequent surgical procedure within 30 days of the initial operation for any reason; planned reoperations and procedures unrelated to the initial surgery were excluded [12]. Readmission was defined as an unplanned return to the hospital within 30 days of discharge for any reason; planned readmissions, transfers to other facilities, and deaths were excluded [13]. Conversion to open was defined as the change from a robotic or laparoscopic ileocolic resection to an open ileocolic resection owing to any intraoperative difficulty or complication [19]. In addition, data concerning the anastomotic configuration used during the operation was collected, which included intracorporeal anastomosis, Kono-S anastomosis, S–S isoperistaltic anastomosis, S–S antiperistaltic anastomosis, and whether a loop ileostomy was constructed.

## Results

### Study selection, characteristics, and risk of bias

**Fig. 1:**
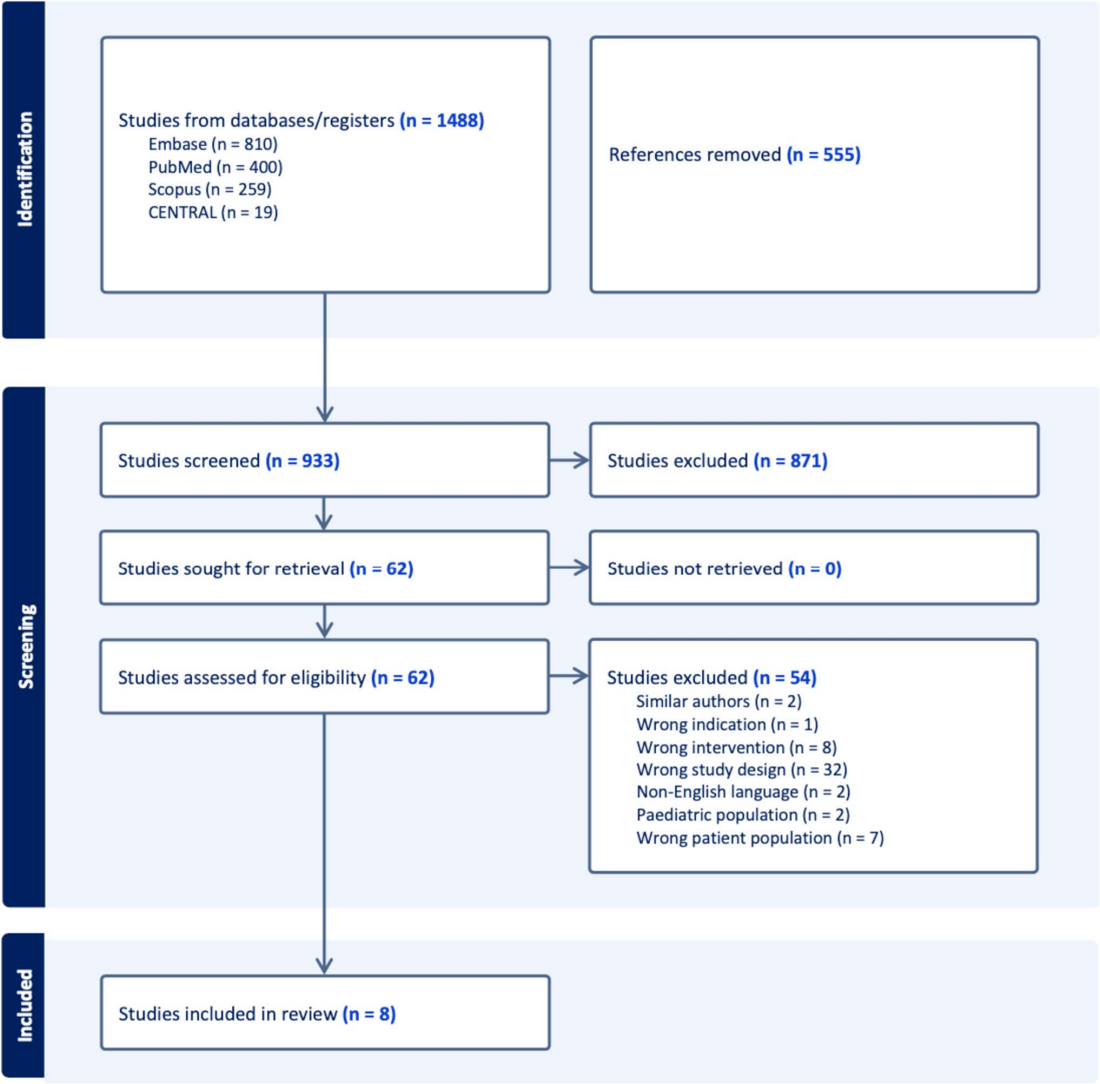
PRISMA flow diagram

The initial search yielded 1488 potentially relevant articles, of which eight unique studies met the inclusion criteria, including 5760 patients that underwent a total of 369 robotic procedures, 3467 laparoscopic procedures, and 1924 open ileocecal resections [15–22]. The details of the study selection process and PRISMA flow diagram are shown in Fig. 1**.**

**Fig. 2:**
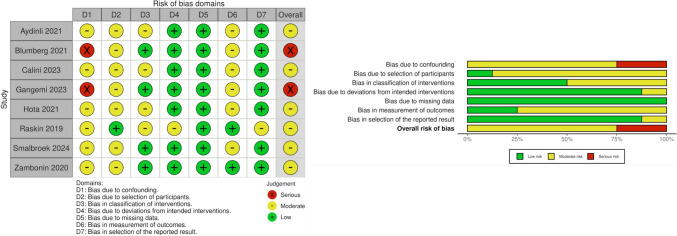
Risk of Bias Assessment —ROBINS-I

Results of the quality assessment of all the included studies are shown in Fig. 2. Of the included studies, two studies were judged to have a serious risk of bias [15, 16], while six studies were judged to have a moderate risk of bias [17–22]. The studies assessed as having a serious risk of bias were owing to an insufficient adjustment for confounding factors, small sample sizes, and limitations in study design and reporting, which reduced the reliability and generalizability of their findings [15, 16]. The remaining six studies [17–22] were rated as having a moderate risk of bias owing to retrospective designs, incomplete adjustment for confounders, and minor issues with data reporting or missing information. These limitations were not deemed substantial enough to compromise the overall validity of their results.

### Baseline and clinical characteristics

The baseline characteristics of 5760 patients who underwent ileocolic resection for Crohn’s disease are presented in Table 1. In total, 369 out of 5760 (6.41%) patients underwent robotic surgeries; 260 (58.20%) of the patients were female [95% confidence interval (CI): 0.50, 0.66; *I*^2^ = 21.84%, *P* = 0.26] and the mean age of the participants was 37.78 years (95% CI: 33.86, 41.70; *I*^2^ = 74.49%, *P* < 0.001). The mean baseline body mass index (BMI) was 26.78 (95% CI: 23.37, 30.19; *I*^2^ = 95.48%, *P* < 0.001) [15–19, 21]. Among the patients, 32 (27.80%) had previous abdominal surgery (95% CI: 0.16, 0.44; *I*^2^ = 58.13%, *P* = 0.07) [16–19].

**Table 1:**
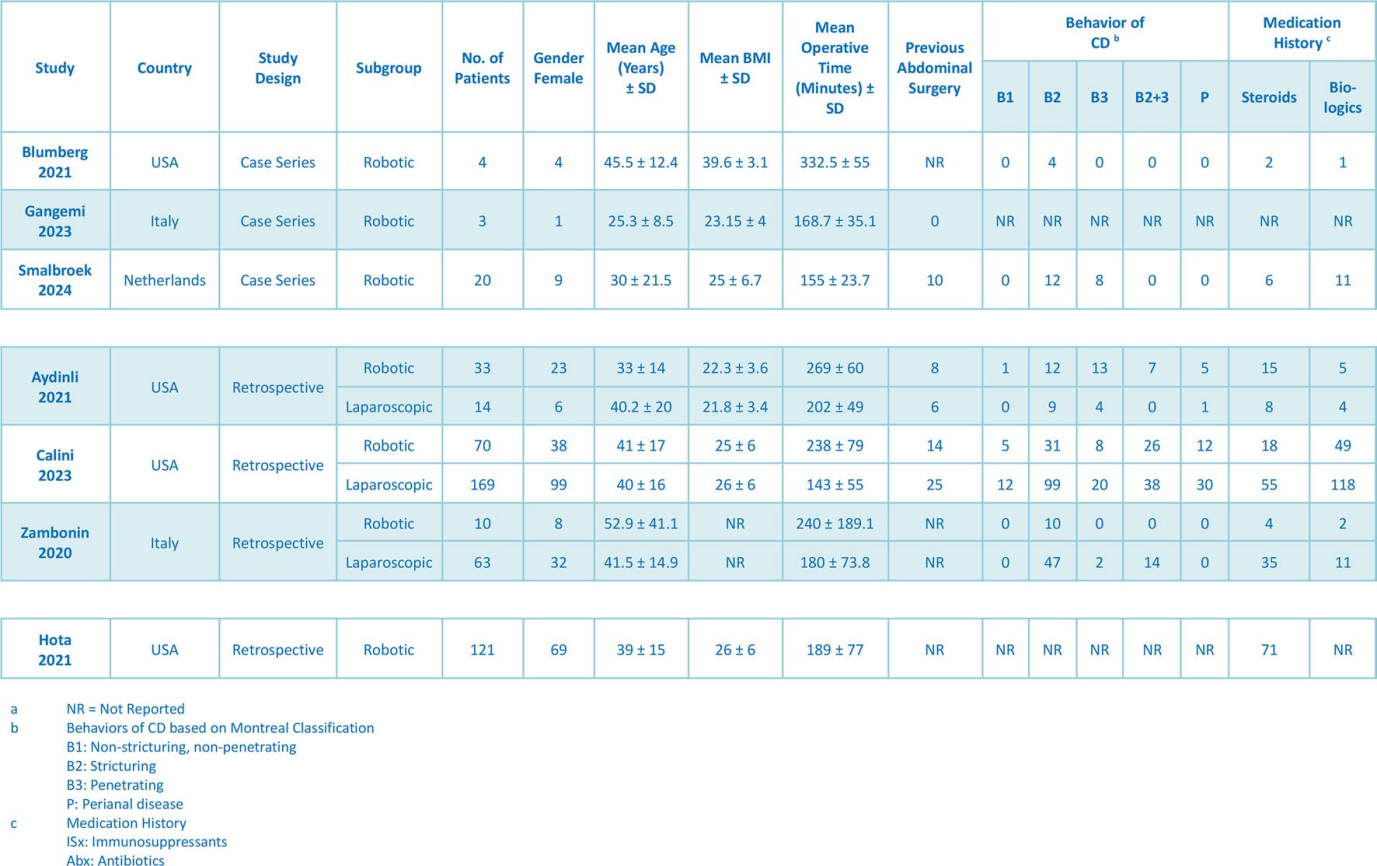
Baseline characteristics of included studies and patients

The characteristics of Crohn’s disease were determined in 137 patients undergoing robotic surgery. In the studies, 6 (5.90%) of the patients had B1 disease (95% CI: 0.03, 0.12; *I*^2^ = 0.00%, *P* = 0.86), 69 (53.70%) had B2 disease (95% CI: 0.36, 0.71; *I*^2^ = 60.80%, *P* = 0.04), 33 (18.70%) had B2 + 3 disease (95% CI: 0.08, 0.38; *I*^2^ = 60.03%, *P* = 0.04), 29 (22.20%) had B3 disease (95% CI: 0.10, 0.43; *I*^2^ = 72.50%, *P* = 0.06), and 17 (14.90%) had P disease (95% CI: 0.10, 0.22; *I*^2^ = 0.00%, *P* = 0.55) [15, 17–20]. Among the patients, 68 (37.30%) had a history of biologics use (95%CI: 0.16, 0.66; *I*^2^ = 85.16%, *P* < 0.001) and 116 (40.90%) had a history of steroid use (95% CI: 0.27, 0.56; *I*^2^ = 75.79%, *P* < 0.001).

### Outcomes of robotic procedure

The characteristics of all the procedures are comprehensively presented in Table 2. The mean total operative time of the robotic procedures was 226 min (95% CI: 189.01, 262.54; *I*^2^ = 96.29%, *P* < 0.001) [14–22]. For the robotic procedures, 123/127 (93.60%) used the intracorporeal anastomosis technique (95% CI: 0.67, 0.99; *I*^2^ = 70.12%, *P* = 0.02) [15, 17–19]. Kono-S anastomosis was performed in 20/103 (17.40%) of the robotic procedures (95% CI: 0.01, 0.90; *I*^2^ = 85.29%, *P* < 0.001), while S–S isoperistaltic anastomosis was performed in 73/103 (50.80%) (95% CI: 0.05, 0.96; *I*^2^ = 86.67%, *P* < 0.001), and S–S antiperistaltic anastomosis was performed in 10/103 (13.50%) (95% CI: 0.02, 0.51; *I*^2^ = 66.36%, *P* = 0.03) [16, 17, 19, 20]. A loop ileostomy was constructed in 10/241 (5.30%) of patients (95% CI: 0.03, 0.11; *I*^2^ = 27.04%, *P* = 0.24) [17–20, 22]. In total, 121/369 (25.50%) complications occurred in the robotics group (95% CI: 0.16, 0.39; *I*^2^ = 76.42%, *P* < 0.001) [16, 18, 19, 22].

**Table 2:**
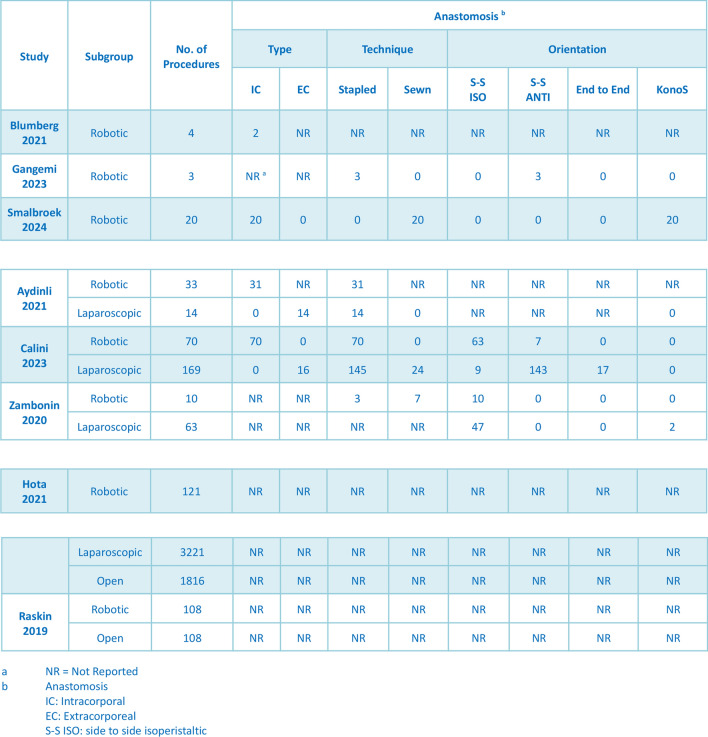
Procedural characteristics

**Fig. 3:**
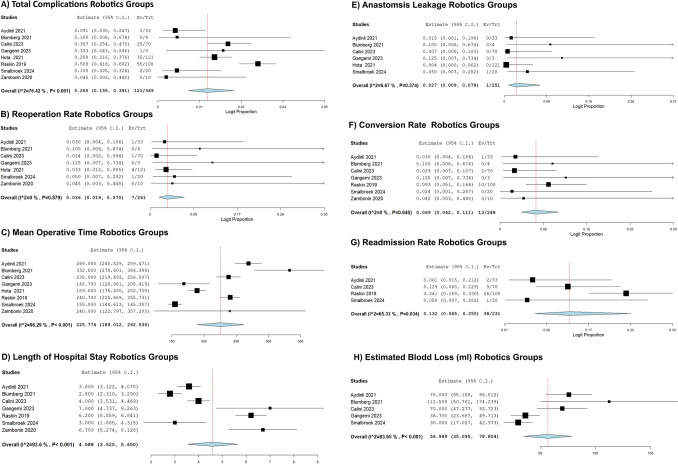
Forest plot one-arm analysis for robotics groups. **A** Intraoperative complications, **B** reoperation rate, **C** mean operative time, **D** length of stay, **E** anastomotic leakage, **F** conversion rate, **G** readmission rate, **H** estimated blood loss

The postoperative data for all the procedures are comprehensively described in Table 3. For the robotic groups, the mean estimated blood loss (EBL) was 56.95 mL (95% CI: 35.10, 78.80; *I*^2^ = 83.65%, *P* < 0.001) [15–19], and the mean length of hospital stay was 4.59 days (95% CI: 3.53, 5.65; *I*^2^ = 93.60%, *P* < 0.001) [15–20, 22]. Out of 369 patients who underwent robotic surgery, 95 surgical and 22 medical complications were recorded. The most common surgical complication was postoperative ileus, occurring in 43/369 (12.50%) of the patients (95% CI: 0.09, 0.16; *I*^2^ = 0.00%, *P* = 0.70), followed by wound complications in 26/369 (7.00%) (95% CI: 0.04, 0.13; *I*^2^ = 37.29%, *P* = 0.13), and postoperative bleeding in 12/369 (4.00%) (95% CI: 0.01, 0.11; *I*^2^ = 40.56%, *P* = 0.11) [15–22]. Anastomotic leakage occurred in 1/251 (2.70%) of the patients (95% CI: 0.01, 0.08; *I*^2^ = 6.67%, *P* = 0.37) [15–19, 21]. Other complications (*Clostridium difficile* infection, intestinal obstruction, intra-abdominal abscess, etc.) occurred in 14/369 (4.50%) of the patients (95% CI: 0.03, 0.07; *I*^2^ = 0.00%, *P* = 0.57) [15–22]. Medical complications occurred in 22/369 (7.10%) of the patients (95% CI: 0.04, 0.12; *I*^2^ = 27.66%, *P* = 0.21) [15–22]. Reoperation was reported in 7/261 (3.60%) of the patients (95% CI: 0.02, 0.07; *I*^2^ = 0.00%, *P* = 0.88) [15–21], and readmissions were reported in 38/231 (13.2%) (95% CI: 0.07, 0.25; *I*^2^ = 65.33%, *P* = 0.03) [17–19, 22]. The surgery was converted to open in 13/248 (6.90%) of the patients (95% CI: 0.04, 0.11; *I*^2^ = 0.00%, *P* = 0.64) [15–20, 22]. Forest plots of the one-arm analysis on total complications, reoperation rate, operative time, length of stay, anastomotic leakage, conversions, readmissions, and estimated blood loss for robotics groups are shown in Fig. 3**.**

**Table 3:**
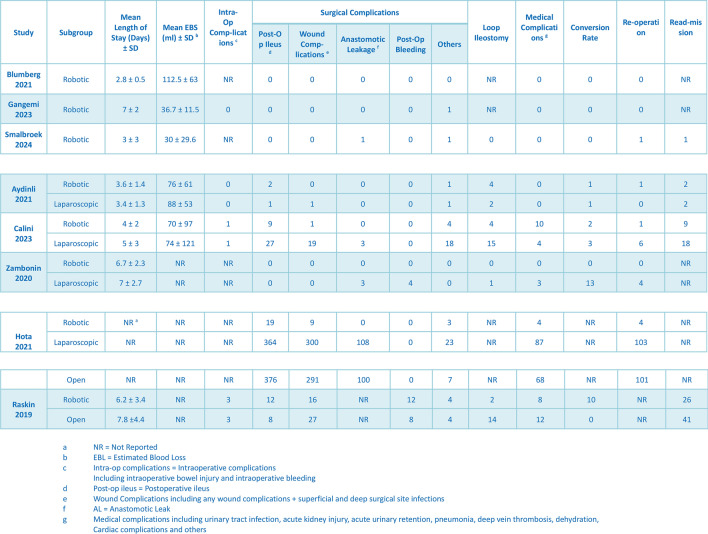
Postoperative outcomes and complications

### Robotic versus laparoscopic procedures

**Fig. 4:**
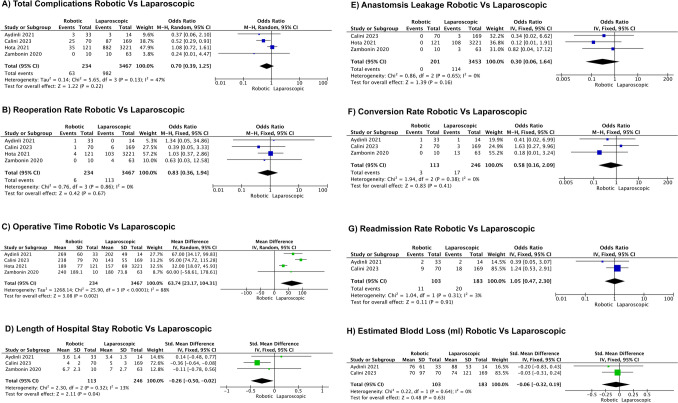
Forest plots of two-arm analysis for robotic versus laparoscopic procedures. **A** Total complications robotic (R) versus laparoscopic (L), **B** reoperation rate R versus L, **C** operative time R versus L, **D** length of stay R versus L, **E** anastomotic leakage R versus L,**F** conversion rate R versus L, **G** readmission rate R versus L, **H** estimated blood loss R versus L

**Fig. 5:**
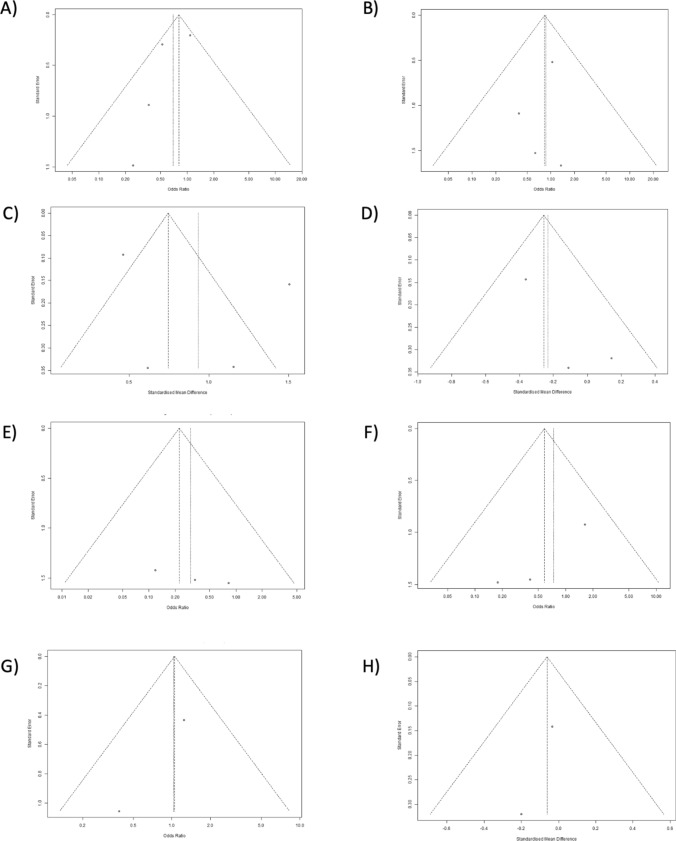
Funnel plots of two-arm analysis for robotic versus laparoscopic procedures. **A** Total complications R versus L, **B** reoperation rate R versus L, **C** operative time R versus L, **D** length of stay R versus L, **E** anastomotic leakage R versus L, **F** conversion rate R versus L, **G** readmission rate R versus L, **H** estimated blood loss R versus L

Robotic procedures had a longer operative time (OR = 63.74, 95% CI: 23.17, 104.31; *I*^2^ = 88.00%, *P* = 0.002) [18–21]. Reoperation rates were similar between robotic and laparoscopic procedures (OR = 0.83, 95% CI: 0.36, 1.94; *I*^2^ = 0.00%, *P* = 0.67) [18–21], as were conversion rates (OR = 0.58, 95% CI: 0.16, 2.09; *I*^2^ = 0.00%, *P* = 0.41) [18–20], rates of postoperative ileus (OR = 1.19, 95% CI: 0.78, 1.82; *I*^2^ = 0.00%, *P* = 0.43) [18–21], total complications (OR = 0.70, 95% CI: 0.39, 1.25; *I*^2^ = 47.00%, *P* = 0.22), estimated blood loss (EBL) (OR =  – 0.06, 95% CI: –0.32, 0.19; *I*^2^ = 0.00%, *P* = 0.63) [18, 19], and anastomotic leak (OR = 0.30, 95% CI: 0.06, 1.64; *I*^2^ = 0.00%, *P* = 0.16). Readmission rates were also comparable between robotic and laparoscopic procedures (OR = 1.05, 95% CI: 0.47, 2.30; *I*^2^ = 3.00%, *P* = 0.91) [18, 19]. Length of stay (LOS) favored the robotic group (OR =  –0.26, 95% CI: –0.50, –0.02; *I*^2^ = 13.00%, *P* = 0.04) [18–20]. Forest plots of two-arm analysis are shown in Fig. 4. Funnel plots of two-arm analysis for robotic versus laparoscopic procedures are shown in Fig. 5**.**

### Robotic versus open procedures

**Fig. 6:**
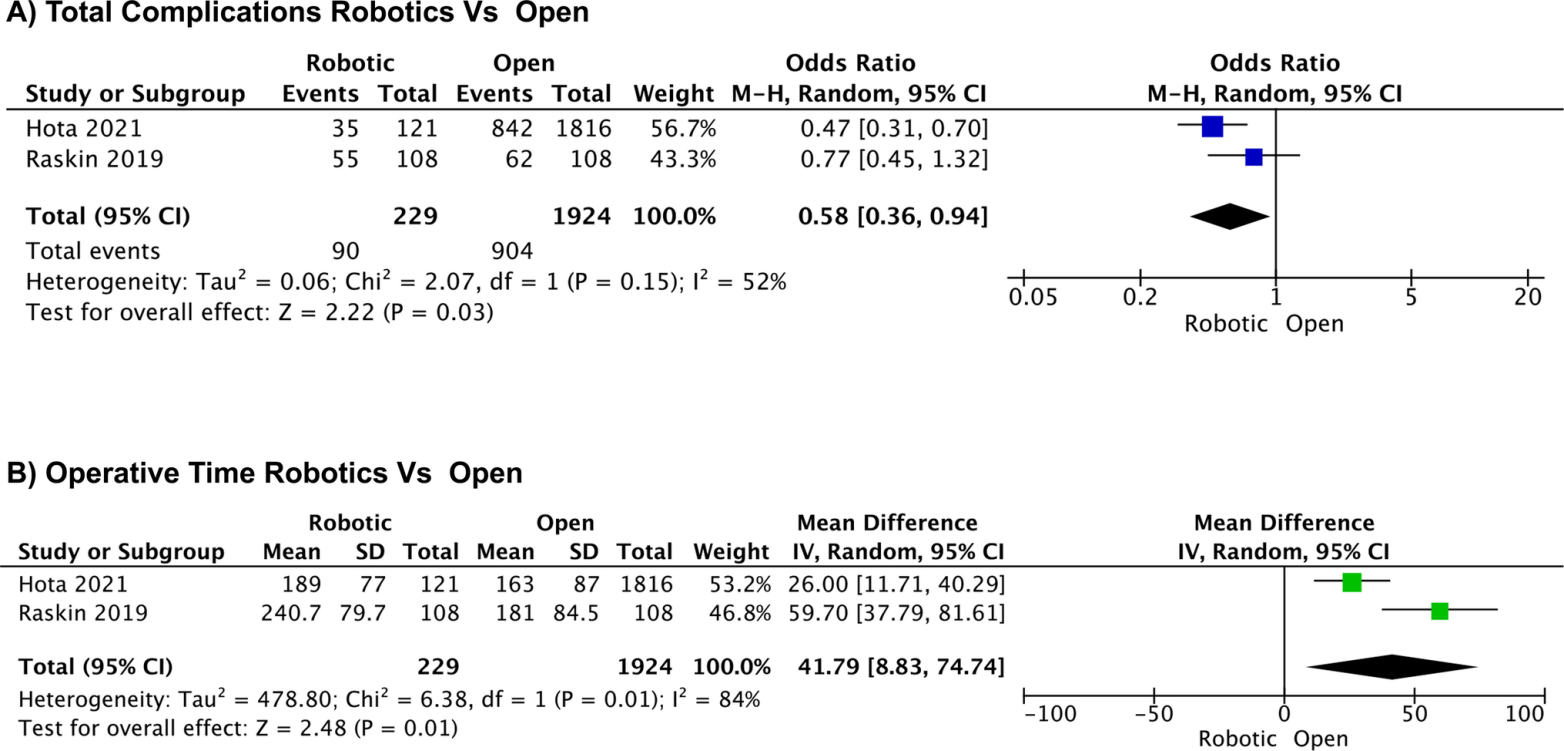
Forest plots of two-arm analysis for robotic versus open procedures. **A** Total complications R versus open (O), **B** operative time R versus O

**Fig. 7:**
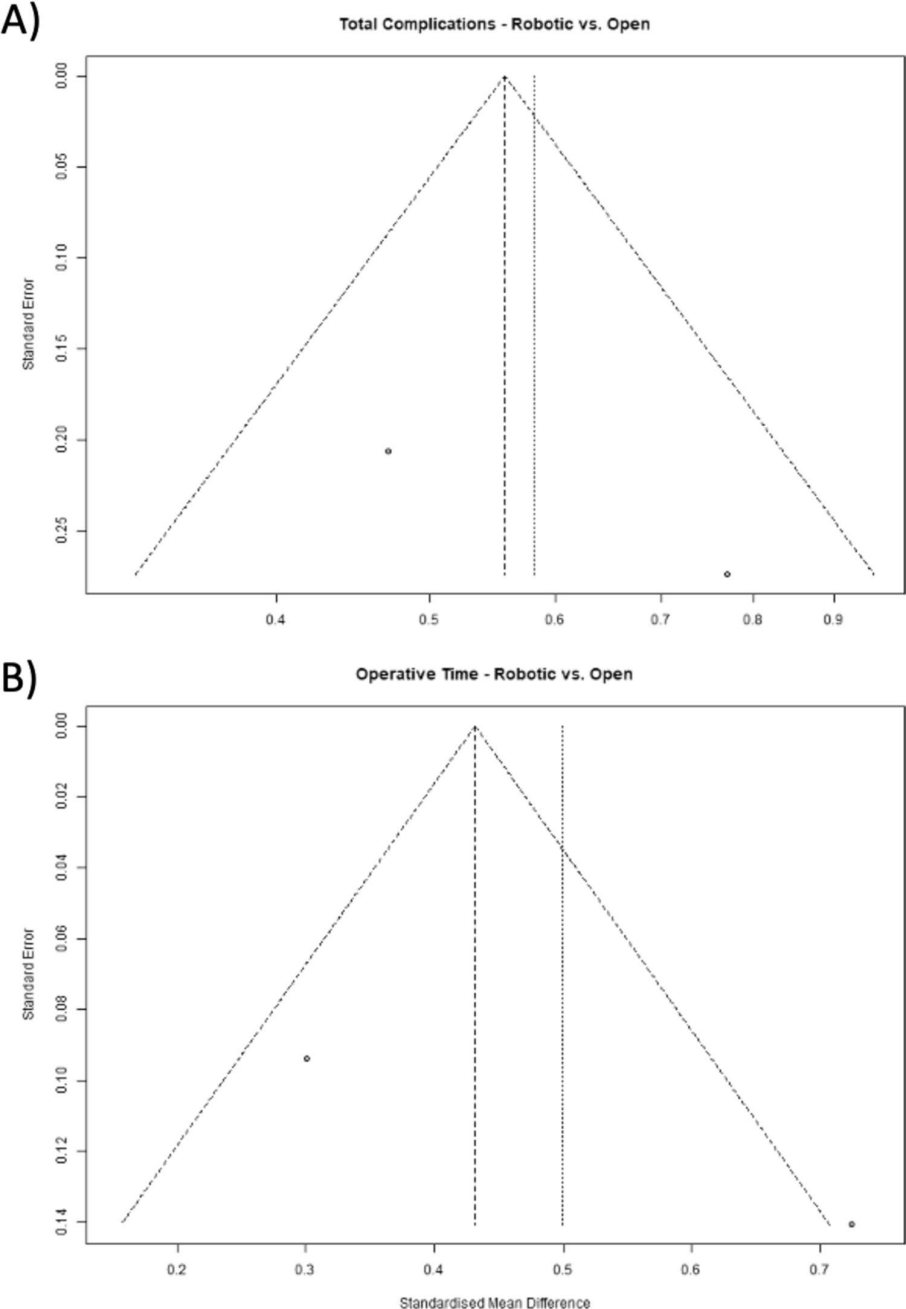
Funnel plots of two-arm analysis for robotic versus open procedures. **A** Total complications R versus O, **B** operative time R versus O

The outcomes of open procedures were assessed in two studies [21, 22]. The robotic procedures showed favorable outcomes compared with open procedures in terms of the total postoperative complication rates (OR = 0.58, 95% CI: 0.36, 0.94; *I*^2^ = 52.00%, *P* = 0.03) [21, 22]. Operative time was analyzed in two studies. The pooled estimate of operative time demonstrates that robotic procedures had a longer operative time than open procedures (OR = 41.79, 95% CI: 8.83, 74.74; *I*^2^ = 84.00%, *P* = 0.01) [21, 22]. Forest plots of the two-arm analysis of the total complications and operative time for robotic versus open studies are shown in Fig. 6. In addition, forest plots of the two-arm analysis for robotic versus open procedures are shown in Fig. 7.

## Discussion

It is a common practice to adopt conservative approaches to surgical resection in IBD, knowing it often necessitates repeated surgery for recurrent disease, particularly in young patients [5]. Repeated open surgery leads to intra-abdominal adhesions and a scarred abdominal wall, which contributes to significant morbidity and risk during future surgery [5]. Hence, laparoscopic surgery has gained popularity and has become the preferred approach as a safe surgical strategy in primary and complicated cases of Crohn’s disease [5]. A challenge surgeons face is conducting laparoscopic procedures within a confined space, which can be difficult to overcome and requires surgeon experience and skills. Laparoscopic surgery has limitations owing to rigid instruments, two-dimensional visualization, and difficulty performing intracorporeal suturing; thus, it is common to use extracorporeal anastomosis in laparoscopic colectomies. Using robotic surgery in these cases offers advantages and helps overcome these challenges by utilizing wristed instruments and better and more precise visualization of the operation site, enabling precise intracorporeal anastomosis. This, in turn, allows for smaller off-midline extraction sites, thus improving cosmetic outcomes and reducing incisional hernia rates [23]. Robotic procedures can overcome these limitations by providing three-dimensional images, enabling more surgeons to participate in minimally invasive surgery with less effort than traditional laparoscopic surgery [23].

Our study showed that robotic surgery has similar safety and efficacy profiles to laparoscopic procedures, with longer operative times but shorter hospital stays, and it showed favorable postoperative complication rates compared with open procedures. The mean operative times for the robotic procedures were longer than the laparoscopic or open procedures. This can be attributed to instrument setup and docking, which consume a significant portion of the overall time [24]. A study reported that robotic surgery takes an average of 41.5 min longer than laparoscopic surgery [25]. In addition, variability in operative times can be influenced by differences in the experience level among surgeons performing these procedures and the experience of the operative staff [26]. However, excluding the preparation time, the operative times between laparoscopic and robotic surgery were relatively similar [27]. Moreover, faster learning curves were observed with robotic surgery and a subsequent drop in the operative times [28]. However, in our study, we were not able to account for different learning curves and surgeon experience, which increase the risk of confounding bias; thus, long-term standardized studies are needed.

Medical and surgical complications associated with robotic surgery were relatively minimal, with low heterogeneity across the different complication types. The most common surgical complication was postoperative ileus. These findings suggest a consistent and favorable safety profile for robotic ileocolic resection. The robotic postoperative complication rates were comparable to the laparoscopic group but significantly lower compared with the open group. However, the sample size of the laparoscopic group in our study was significantly higher than the robotic group, thus increasing the risk of sampling bias and decreasing the power of the study. Nonetheless, our findings are consistent with numerous other studies that have reported lower complication rates for robotic surgery compared with open surgery [29].

Moreover, our results are consistent with literature showing that the length of hospital stay was significantly shorter in the robotic group compared with the laparoscopic group [30]. This could be attributed to the shorter duration it takes patients to regain bowel function, as observed in robotic surgery [31]. Although we did not see a difference in postoperative ileus between robotic and laparoscopic surgery, the granularity of the data in this meta-analysis regarding the return of bowel function is limited to calculate and quantify any difference.

Prior studies consistently highlight the significantly higher expenses associated with robotic procedures [32, 33]. Conducting a cost–benefit analysis for robotic ileocolic resection requires an inclusive evaluation of the costs and the potential clinical advantages. Future analysis should consider a comprehensive assessment of both direct and indirect expenses, such as equipment, maintenance, and training, as well as shorter hospital stays and reduced complication rates. Although our meta-analysis demonstrates the clinical advantage of shorter hospital stays with robotic ileocolic resection compared with laparoscopic, and lower complication rates in comparison to open surgery, the resulting hospital savings need to be balanced against the additional cost of equipment and operating time. Thus, more research is still needed to assess the cost-effectiveness and clinical benefit of robotic surgery.

This study is the first reported meta-analysis assessing the safety and efficacy of robotic ileocolic resection for Crohn’s disease. It also compares all three surgical modalities to provide better insights into the benefits and drawbacks of each procedure. To ensure inclusivity and accuracy, there was no time constraint during the initial screening process. However, the study has several limitations. First, all included studies were retrospective or case series, which carry a higher risk of selection bias. Only eight studies were included in the analysis, and many data points of interest were not recorded across all studies, therefore limiting the ability to assess publication bias and reducing generalizability. In addition, the study did not assess the learning curve for robotic ileocolic resection or account for the skill of the surgeon, potentially introducing bias in the results. Our review focused only on short-term outcomes (30 days), including postoperative complications, as there are limited studies assessing the long-term outcomes of robotic surgery. Moreover, several factors in these studies acted as confounding factors affecting the outcome of the surgery, including the type of anastomosis technique and patient factors such as BMI. In our study, several anastomotic techniques were used, including stapled side-to-side anastomosis, Kono-S technique, and hand-sewn end-to-end anastomosis, which introduces bias and confounding to our results. In addition, only one study included patients with a BMI higher than 30, which could potentially cause an overestimation of the benefits of robotic surgery, as patients with obesity have an increased risk of technical challenges and complications associated with performing robotic surgery [34, 35]. Thus, further large, randomized studies comparing robotic, laparoscopic, and open surgeries are warranted in this patient population.

## Conclusions

This meta-analysis suggests the potential benefits of robotic surgery for ileocolic resection in Crohn’s disease, demonstrating comparable safety and efficacy to laparoscopic procedures. Despite longer procedure time, robotic surgery offers advantages such as a shorter hospital stay and lower postoperative complication rates compared with open surgery. The study highlights the consistent safety profile of robotic procedures and their capability to mitigate the challenges faced in traditional laparoscopic surgery. However, the findings are limited by the retrospective nature of the included studies, potential biases, and the small sample size of robotic procedures. Future research should focus on larger, randomized studies to validate these findings and explore the long-term outcomes and cost-effectiveness of robotic surgery for Crohn’s disease.

## Supplementary Information

Below is the link to the electronic supplementary material.

Supplementary file1 (PDF 84 KB)

## Data Availability

Data are provided within the manuscript or supplementary information files. With the publication, the data set used for this meta-analysis can be shared upon request from the study authors.

## Abbreviations

BMI: Body mass index
CI: Confidence interval
*C. diff*: *Clostridium difficile*
EBL: Estimated blood loss
*I*^2^: Heterogeneity index
IBD: Inflammatory bowel disease
LOS: Length of stay
OR: Odds ratio
PROSPERO: International Prospective Register of Systematic Reviews
PRISMA: Preferred Reporting Items for Systematic Reviews and Meta-Analyses
ROBINS-I: Risk of Bias in Non-randomized Studies of Interventions
SD: Standard deviation
